# Supplementary material for: Gabapentin and Duloxetine Prevent Oxaliplatin- and Paclitaxel-Induced Peripheral Neuropathy by Inhibiting Extracellular Signal-Regulated Kinase 1/2 (ERK1/2) Phosphorylation in Spinal Cords of Mice
Source: Pharmaceuticals (Basel). 2020 Dec 31;14(1):30. doi: 10.3390/ph14010030 (PMC7824557; doi:10.3390/ph14010030)

Supplementary Figure

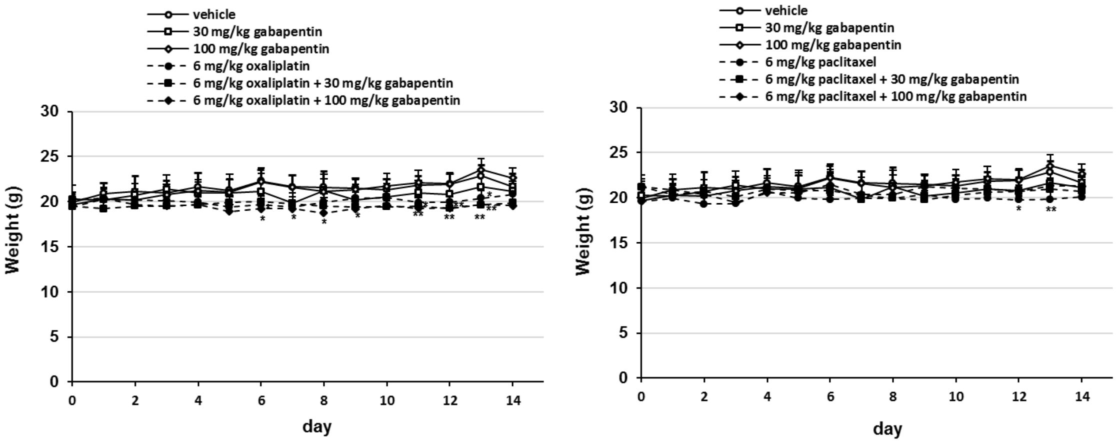

Figure S1. Safety of oxaliplatin, paclitaxel, and gabapentin administrated in vivo. Oxaliplatin (6 mg/kg, i.v.) and paclitaxel (6mg/kg, i.v.) were administered on days 0 and 7. Gabapentin (30 or 100 mg/kg, p.o.) was administered daily from days 0 to 14. Mice were weighed before the first treatment and daily throughout the treatment period. Data are presented as means and S.D. *P < 0.05, **P < 0.01 vs. vehicles (Shapiro-Wilk test and one-way analysis of variance (ANOVA) with Tukey test).

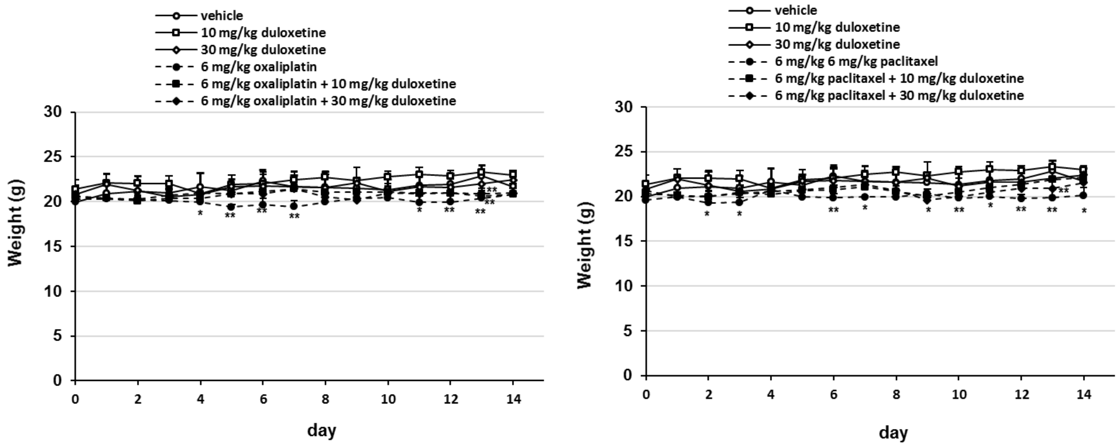

Figure S2. Safety of oxaliplatin, paclitaxel, and duloxetine administrated in vivo. Oxaliplatin (6 mg/kg, i.v.) and paclitaxel (6mg/kg, i.v.) were administered on days 0 and 7). Duloxetine (10 mg/kg or 30mg/kg, p.o.) was administered daily from days 0 to 14. Mice were weighed before the first treatment and daily throughout the treatment period. Data are presented as means and S.D. *P < 0.05, **P < 0.01 vs. vehicles (Shapiro-Wilk test and one-way analysis of variance (ANOVA) with Tukey test).

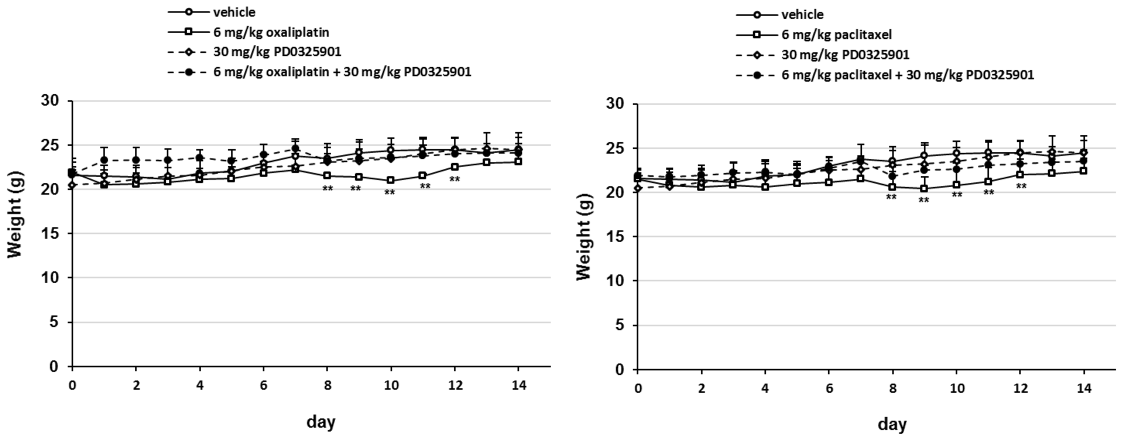

Figure S3. Safety of oxaliplatin, paclitaxel, and PD0325901 administrated in vivo. Oxaliplatin (6 mg/kg, i.v.) and paclitaxel (6mg/kg, i.v.) were administered on days 0 and 7). PD0325901 (30mg/kg, p.o.) was administered daily from days 0 to 14. Mice were weighed before the first treatment and daily throughout the treatment period. Data are presented as means and S.D. *P < 0.05, **P < 0.01 vs. vehicles (Shapiro-Wilk test and one-way analysis of variance (ANOVA) with Tukey test).

Figure 5

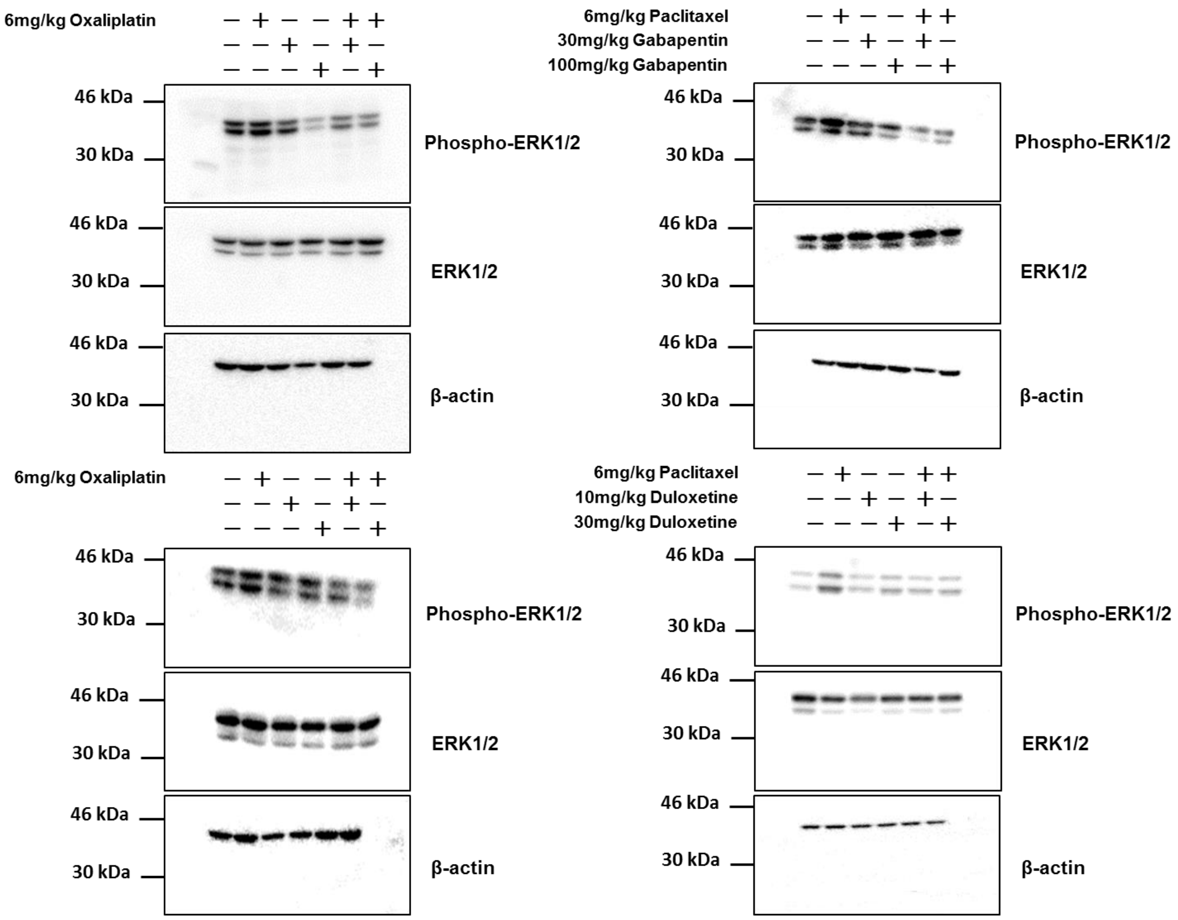

Figure 8

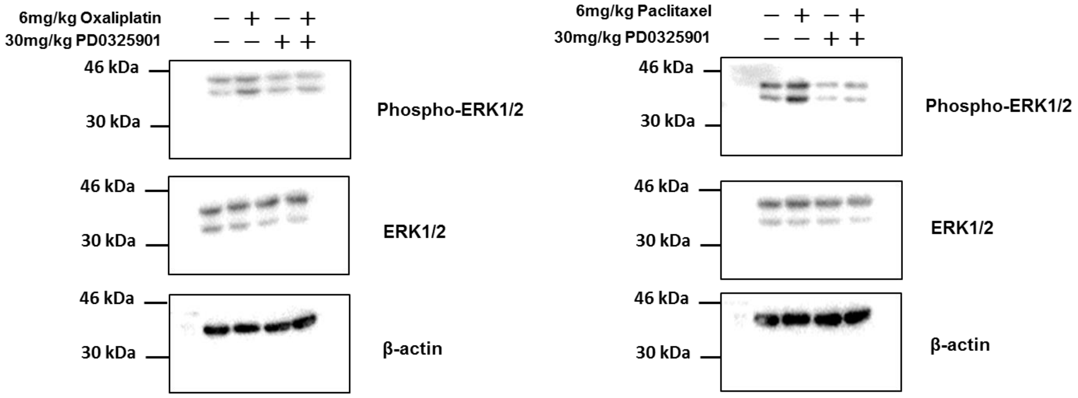

Supplement: Supplementary file 1 [file pharmaceuticals-14-00030-s001.pdf]
